# Topics of the Month

**Published:** 1895-07

**Authors:** 


### TOPICS OF THE MONTH.

AROUND the American Medical Association, at Baltimore, clustered a group of meetings that were of more importance even than the association itself. Of these we may mention, first, the American Academy of Medicine. The meetings of this body are increasing year by year in importance and in influence. At this one, Dr. Leartus Connor, of Detroit, read a paper entitled, Drifting—Who, how, whither? that deserves to be widely read. The *Cleveland Medical Gazette* for June gives an interesting synopsis of this paper, from which we extract the following:

> It is believed that the profession is drifting toward the adoption of a law of consultations somewhat as follows: "Every physician shall be deemed eligible for professional consultation who has shown that he has had such preliminary training as enabled him to comprehend the study of medicine; has fully mastered the elements of medical science and art; has complied with existing laws respecting physicians, and who maintains an honorable reputation in the community in which he resides." Of these qualifications the physicians of his locality shall be the final judges. If these, who know him best, shall indorse him, then shall he be freely admitted to medical organizations and be eligible for consultations.

---

Another important one of this group of meetings was that of the American Medical College Association, whose action in extending the curriculum from three to four years we referred to in our last issue. Nearly all the Southern colleges applied for membership. It was stated that but two Northern colleges in affiliation with the association would enter upon less than a four years' course during the next academic year.

Dr. William Osler, of Baltimore, was elected president of the association, and Dr. Bayard Holmes, of Chicago, secretary. Drs. H. P. Bowditch, of Boston; Brigadier-General George M. Sternberg, Surgeon-General U. S. Army; T. Gaillard Thomas, of New York; N. S. Davis, of Chicago; and R. Beverly Cole, of San Francisco, were elected honorary members.

---

The Association of American Medical Editors, too, held its annual meeting on Monday, May 6, 1895, and elected the following officers: President, Dr. George M. Gould, Philadelphia; treasurer, Dr. A. H. Ohman-Dumesnil, St. Louis; secretary, Dr. H. B. Ellis, Los Angeles, Cal. The annual banquet was held on Monday evening, in which the Medical Publishers' Association joined. Dr. I. N. Love, editor of the St. Louis *Medical Mirror*, performed the functions of toastmaster with his usual skill and ability.

